# Sonochemical Reaction of Bifunctional Molecules on Silicon (111) Hydride Surface

**DOI:** 10.3390/molecules26206166

**Published:** 2021-10-13

**Authors:** Serge Ismael Zida, Yue-Der Lin, Yit Lung Khung

**Affiliations:** 1 Ph.D. Program of Electrical and Communications Engineering, College of Information and Electrical Engineering, Feng Chia University, No.100 Wenhwa Road, Seatwen, Taichung 40724, Taiwan; p0766289@o365.fcu.edu.tw (S.I.Z.); ydlin@fcu.edu.tw (Y.D.L.); 2 Department of Automatic Control Engineering, Feng Chia University, No.100 Wenhwa Road, Seatwen, Taichung 40724, Taiwan; 3 Department of Biological Science and Technology, China Medical University, No.100 Jingmao 1st Road, Beitun District, Taichung City 406, Taiwan

**Keywords:** sonochemical reaction, sonochemistry, acoustic cavitation, surface grafting, nucleophilic reaction

## Abstract

While the sonochemical grafting of molecules on silicon hydride surface to form stable Si–C bond via hydrosilylation has been previously described, the susceptibility towards nucleophilic functional groups during the sonochemical reaction process remains unclear. In this work, a competitive study between a well-established thermal reaction and sonochemical reaction of nucleophilic molecules (cyclopropylamine and 3-Butyn-1-ol) was performed on *p*-type silicon hydride (111) surfaces. The nature of surface grafting from these reactions was examined through contact angle measurements, X-ray photoelectron spectroscopy (XPS) and atomic force microscopy (AFM). Cyclopropylamine, being a sensitive radical clock, did not experience any ring-opening events. This suggested that either the Si–H may not have undergone homolysis as reported previously under sonochemical reaction or that the interaction to the surface hydride via a lone-pair electron coordination bond was reversible during the process. On the other hand, silicon back-bond breakage and subsequent surface roughening were observed for 3-Butyn-1-ol at high-temperature grafting (≈150 °C). Interestingly, the sonochemical reaction did not produce appreciable topographical changes to surfaces at the nano scale and the further XPS analysis may suggest Si–C formation. This indicated that while a sonochemical reaction may be indifferent towards nucleophilic groups, the surface was more reactive towards unsaturated carbons. To the best of the author’s knowledge, this is the first attempt at elucidating the underlying reactivity mechanisms of nucleophilic groups and unsaturated carbon bonds during sonochemical reaction of silicon hydride surfaces.

## 1. Introduction

Silicon is an important industrial element for modern transistors [1,2] as well as being of significant importance to the scientific community in the areas of biotechnology applications [3,4] and in biosensing [5,6,7]. In surface chemistry, the formation of stable Si–C linkages on silicon surfaces results in highly resilient surfaces that can withstand harsh conditions [8,9]. These surfaces are deemed important in various fields such as in electronics [10,11,12], semiconductors [13,14], and biosensors [15,16]. Thus far, the literature is rich with various hydrosilylation methodologies ranging from transition metal catalysis [17] to thermal [18,19], ultraviolet initiation [20,21], microwaves activation [22,23] and sonochemical reaction [24], to achieve the formation of stable monolayers on silicon surfaces. Despite being widely reported in literature, these methodologies may have certain limitations. For example, a long reaction time as well as the use of high temperature during thermal reaction increase the chance of unnecessary oxidation [18,25], while both thermal and ultraviolet strategies can be highly susceptible to surface adventitious carbon contamination [26,27]. Ultraviolet light is also likely to be absorbed and siphoned by aromatic systems when these are the moieties introduced to the silicon surface. Other reaction methods may also have their individual drawbacks. For example, microwave activation, which is very similar to thermal reaction, has a limited depth of penetration into the material [28]. The use of transition metal catalysis could result in difficulty of surface removal of residual catalyst.

Another interesting reaction strategy for grafting monolayer onto a silicon surface is the approach of sonochemical reaction. It can be generally described as the use of ultrasound irradiation to initiate a reaction of a molecule and this process is often performed at a frequency between 15 kHz–10 MHz [29]. During sonochemical reaction on a silicon surface, the dispersive energy of the ultrasound is condensed in the acoustic cavitation which subsequently accelerates the chemical reaction. The basic principle of sonochemical reaction is based on the phenomenon of acoustic cavitation which can be described as the formation, growth and sudden collapse of bubbles induced during sonochemical reaction [29,30,31]. A significant advantage of sonochemical reaction is that such an approach is inexpensive and environmentally friendly [32]. Other merits also include the simplicity of operation and the uniform dispersion of the ultrasound in the baths [33]. The application of sonochemical reaction is quite broad, ranging from materials synthesis [34,35], nanochemistry [36,37,38] to simple cleaning [39,40,41].

In recent years, sonochemical reaction has attracted much interest from surface chemists for the purpose of performing silicon-surface hydrosilylation [24,42]. Notably, Frederick et al., reported the functionalization of organic monolayers of substituted styrenes on silicon hydride surfaces using a range of styrene derivatives [42]. Interestingly, the use of 4-cyanostyrene in their work was of much interest to our group due to the presence of a cyano nucleophilic group that in turn offers an opportunity to examine nucleophiles’ competitiveness and preference during the sonochemical process. In fact, their discussion only suggested that the 4-cyanostyrene reaction to the silicon hydride surface under sonochemical reaction occurred through the alkene group instead of the nucleophile but very limited information has been provided for the reasons behind this preference. Hence, at this point of writing, very little is known about the behavior of nucleophiles during a sonochemical-based reaction and a direct comparison between nucleophiles and unsaturated carbon has not yet been reported in literature.

Thus, to shed light into the reaction mechanisms and the preferential bond directionality between unsaturated carbon and nucleophiles during sonochemical reaction, a comparative analysis between sonochemical reaction and thermal excitation was made to conceptualize the overall reaction route of the cyclopropylamine and 3-Butyn-1-ol molecules under both reaction conditions. Thermal reactions of nucleophile were included in this comparative study because our group had previously reported in many accounts the specific preference of nucleophile reacting to silicon hydride surfaces under high temperatures [19,43,44,45]. This would enable a quick comparison between both reaction processes. The choice of cyclopropylamine was based on its radical susceptibility as described by Nonhebel [46], as this provided us with a window to detect for silicon dangling bonds on the surface which may produce a potential ring opening event of cyclopropylamine. In contrast, 3-Butyn-1-ol has an interesting structure with unsaturated carbon bonds at one end and the hydroxide group at the other end. This structure was important because it gave an opportunity to directly compare the reaction directionality of the molecule (nucleophilic OH or alkyne) during the sonochemical reaction. In the following parts of this work, the surface chemistry analysis through water contact angle measurements, AFM and XPS will be discussed in detail to provide reaction outcomes accordingly.

## 2. Materials and Methods

In this experiment, P-type silicon (111) (0.001–0.005 Ω cm) was ordered from Semiconductor Wafer, Inc. (Hsinchu, Taiwan). Cyclopropylamine (98%), 3-Butyn-1-ol (97%), Mesitylene (98%), Hydrogen peroxide (30%), Hydrofluoric acid (ACS reagent, 48%) as well as sulfuric acid (99.99%) were acquired from Sigma-Aldrich (St Louis, MO, USA), except where noted, and used with no further purification.

### 2.1. Thermal Reaction

P-type silicon (111) wafers were cut in 10 × 10 mm^2^ and cleaned for 30 min in a piranha solution consisting of 1 volume of 33% hydrogen peroxide and 3 volumes of 95% sulfuric acid. Subsequently, a 10% concentration of each compound (Cyclopropylamine and 3-Butyn-1-ol) was diluted in 5 mL mesitylene solution and then transferred to a Schlenk flask for a 15 times freeze–pump–thaw cycle to degas and remove oxygen. After this process, argon gas was added to the Schlenk flask to prevent oxygen from re-entering the solution. The silicon wafers were further cleaned with deionized water and immersed in an aqueous 10% hydrofluoric acid solution for 30 s before being moved to the Schlenk flask solution. The Schlenk flask was submerged in silicon oil for 18 h at 150 °C. After the high-temperature thermal reaction, the samples were collected, copiously rinsed and sonicated with methanol, ethanol and deionized water prior to further surface examinations.

### 2.2. Sonochemical Reaction

P-type silicon wafers (111) were prepared and freeze–pump–thaw cycles were performed in a similar procedure as for the thermal excitation reaction. After transferring the silicon hydride wafers into the solution, the Schlenk flask was then immersed for 2 h at room temperature in the water bath DELTA^®^ DC150H sonicator (Delta Ultrasonic Co., Ltd., New Taipei, Taiwan) at a frequency of 40 KHz. Once the reaction was completed, the samples were collected, thoroughly rinsed and sonicated with methanol, ethanol and deionized water before surface analysis. The reaction was identical for each of the compounds, cyclopropylamine and 3-Butyn-1-ol.

### 2.3. Contact Angle Measurements

The contact angle measurements were performed at China Medical University in Taichung, Taiwan, immediately after the reactions, and the images were obtained via the High View Innovation (HVI) Contact Angle Analyzer (CA1) (Hsinchu, Taiwan). This equipment had a built-in real-time image acquisition and automatic analysis function with an angle measurement from 0 to 180°. A triplicate measurement was performed with a 2 μL droplet deposited on the surface of each sample while the automatic measurement deviation was <1°. The droplet images were automatically processed and saved using the integrated HVI contact angle software running on a computer with a Windows 10 OS.

### 2.4. Atomic Force Microscopy Measurements (AFM)

The AFM examinations were conducted at Feng Chia University in Taichung, Taiwan, with the Digital Instrument NS4/D3100CL (Karlsruhe, Germany) MultiMode Scanning Probe Microscope software operating on an integrated AFM tapping mode with cantilever. The frequency of operation was 150 kHz while the Force was 5 N/m. Each of the samples was examined at a scan rate of 0.8 Hz with a scan size of 1 × 1 μm. The AFM images were subsequently processed using Gwyddion version 2.59 software running on a Mac OS computer.

### 2.5. X-ray Photoelectron Spectroscopy (XPS)

The XPS measurements were carried out at National Chung Hsing University (NCHU) in Taichung, Taiwan. An XPS PHI 5000 VersaProbe (ULVAC-PHI) (Kanagawa, Japan) instrument fitted with an Al Kα (1486.6 eV) X-ray source was used to perform the analyses. For each of the samples, high-resolution spectra were acquired for C1s, O1s, N1s and Si2p. The deconvolution of the different spectra and the subsequent determination of the full width at half maximum (FWHM) as well as the area under the peak were performed using XPSpeak 4.1 fitting software.

## 3. Results

**Figure 1:**
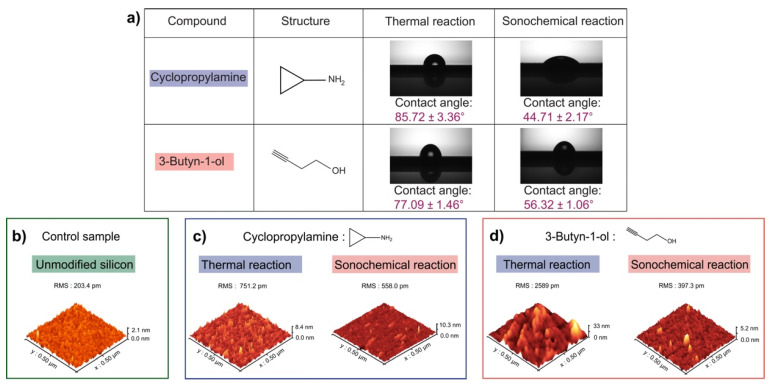
(**a**) Contact angle measurements of cyclopropylamine and 3-Butyn-1-ol after thermal and sonochemical reaction. (**b**) AFM examination of the control sample. (**c**) The AFM characterization of cyclopropylamine showed a roughness value of 751.2 pm for the thermal sample and 558 pm for the sonochemical sample. (**d**) AFM images of 3-Butyn-1-ol after thermal and sonochemical reaction.

Multiple sets of analyses and measurements (triplicates) were conducted after the sample preparation to obtain substantial information to help understand the reactions. Firstly, the outcome of the thermal reactions could be anticipated in view of our previous reports [45,47,48]. More importantly, several questions remained pertaining to how the sonochemical reactions will affect the grafting of the nucleophiles and how the bifunctionality of an alkyne and NH_2_ groups may compete with each other during the sonochemical reaction. Contact angle goniometry of the samples was first performed to observe the wettability of the surfaces, as shown in Figure 1a. The contact angle of cyclopropylamine grafting for thermal excitation showed a wetting angle of 85.72° with a deviation margin of ±3.36°. On the other hand, the water goniometry analysis for the grafting of the same molecule after sonochemical reaction showed an angle of 44.71° with a slight margin of ±2.17°. The significant difference in contact angle noticed from these observations indicated that the reaction proceeded in a dissimilar fashion for both chemical reactions. The high contact angle for cyclopropylamine thermal reaction was as expected and in trend of agreement with our previous work [47] pertaining to grafting cyclopropylamine with thermal reaction. However, the sessile droplet contact angle registering at 44.71° for the sonochemical reaction sample may be indicative of a different reaction profile that may suggest either polymerization [47,49] or non-reactivity, but at this stage of analysis there was not enough details to make a definitive conclusion.

For 3-Butyn-1-ol molecule, the first set of measurements was performed on the thermal reaction sample and the triple sampling had presented a wettability profile of 77.09° ± 1.46°. However, for the sonochemical reaction sample, a lower angle of 56.32° ± 1.06° was noted. As usual, although there was a difference in the wettability profile between sonochemical and thermal reaction, it was not substantial enough to make an assumption at this point. Therefore, additional surface analyses were necessary to understand the possible reaction outcomes and AFM examinations were performed to observe the surface topography as well as to determine the surface roughness for each of the samples.

The AFM examination of the control sample displayed a surface with very little roughness, and the RMS value was approximately 203.4 pm as shown in Figure 1b. Compared with the control sample, some changes were observed on the surface topography of the cyclopropylamine-based thermal reaction with a RMS value of 751.2 pm which, in conjunction with the high contact angle and our past works [19,47], could indicate that a reaction occurred via the nucleophilic group. For the cyclopropylamine sonochemical reaction sample, the previous observation of the low contact angle may have suggested polymerization of the surface. If polymerization did occur, the AFM study should show a thickening layer on the surface. However, such a result was not observed in the AFM examination (as shown in Figure 1c). The RMS value of the sample was 558 pm, and it was interesting to note that this value had tilted towards that of the control sample, but the slight roughening may be explained by the fact that the control sample was not influenced by the etching effect of hydrofluoric acid during the sample preparation.

In Figure 1d, the surface examinations of the sample of 3-Butyn-1-ol after thermal reaction showed a characterized surface roughness, and the RMS value was 2589 pm. In contrast, for the sample of 3-Butyn-1-ol after sonochemical reaction, the RMS decreased significantly to 397.3 pm while significant topographical changes were exhibited on the surface. This low RMS value may be due to the fact that the reaction did not take place or did occur through the formation of a Si–C bond, and the OH group was presented as a distal group, which could also explain the lower contact observed for the sample. Nevertheless, XPS experiments were needed to make further deductions or to support some early hypothesis.

**Figure 2:**
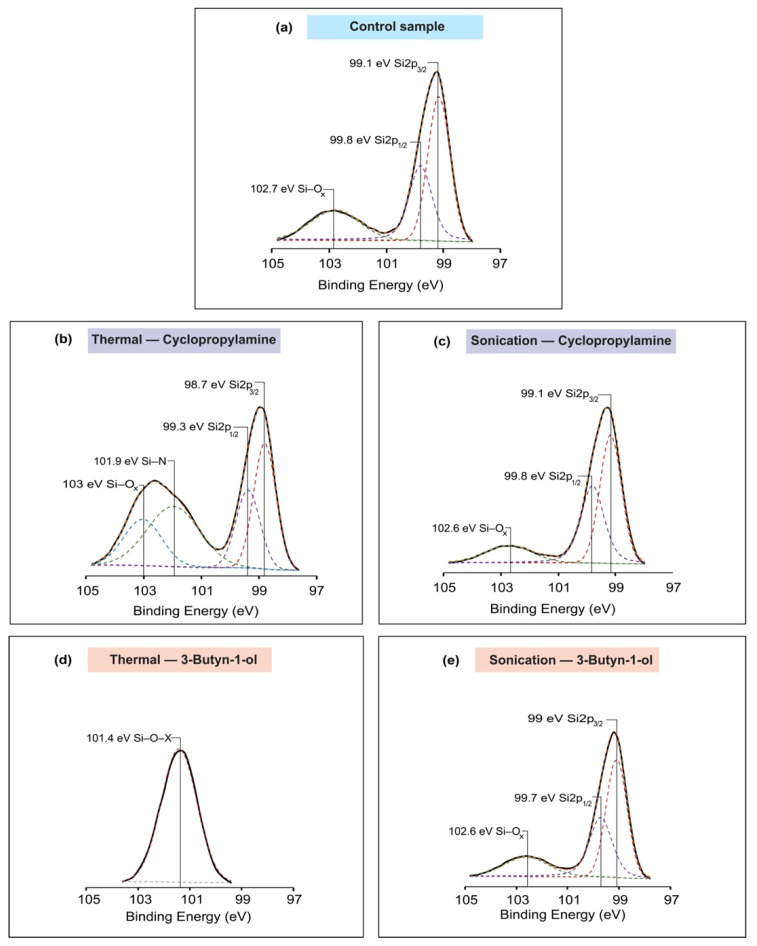
Si2p spectrum deconvolution for (**a**) control sample; (**b**) cyclopropylamine thermal excitation. A silicon nitrogen peak appeared at 101.9 eV; (**c**) cyclopropylamine sonochemical reaction; (**d**) 3-Butyn-1-ol thermal excitation with a single peak fitted at 101.4 eV; (**e**) 3-Butyn-1-ol sonochemical reaction.

XPS studies were carried out to provide more information on the nature of the bond formation mechanism, especially in the presence of nucleophilic groups. As shown in Figure 2a, the analysis of the Si2p spectrum of the control sample indicated 3 peaks at 99.1 eV, 99.8 eV and 102.7, which was, respectively, representative of Si2p_3/2_, Si2p_1/2_ and Si–O_x_ as described previously [50,51]. Furthermore, the Si2p spectrum of the thermal reaction of cyclopropylamine displayed the Si2p_3/2_ and Si2p_1/2_ peaks at 98.7 and 99.3 eV, respectively (as demonstrated in Figure 2b). A peak was fitted at 101.9 eV, which was +3.2 eV shift from the Si2p_3/2_ peak and was typical of Si–N bonding as described by Luo et al. [52] as well as by our previous studies [19,47,48]. The presence of the Si–N bond was indicative that thermal reaction had occurred through the amine group and this was in corroboration with previous accounts [47]. The Si–O_x_ peak was fitted at 103 eV and was a shift of +4.3 eV from the Si2p_3/2_ peak [53,54].

On the other hand, in silicon surfaces that underwent a cyclopropylamine-based sonochemical reaction, the analysis of the spectra did not detect the presence of Si–N linkage. The Si2p_3/2_ and Si2p_1/2_ peaks were observed at 99.1 and 99.8 eV, while a peak at 102.6 eV was attributed to Si–O_x_ as described by Yoon et al. [55]. In comparison with the control sample, it can be noticed that both samples have similar peaks at approximatively the same binding energy position, and this further confirmed that the surface grafting may not have occurred for a cyclopropylamine-based sonochemical reaction.

The XPS deconvolution of the 3-Butyn-1-ol sample for thermal excitation showed a single peak at 101.4 eV that was characteristic of Si–O–X [45]. The presence of a single peak can be explained by the concentration level of 3-Butyn-1-ol (10% concentration in this study). As demonstrated in the past [45], as the concentration of 3-Butyn-1-ol increased, the spectrum tended to lose its Si2p peaks and the reaction proceeded through the nucleophile OH to form Si–O–C bonds at the surface. On the contrary, for the sonochemical reaction, three distinct peaks were successfully fitted during the deconvolution. The Si2p_3/2_, Si2p_1/2_ and Si–O_x_ peaks were observed at 99, 99.7 and 102.6 eV, respectively [55], as shown in Figure 2e. Lower oxidation was detected as compared with the control surface and our attempt to fit a Si–C peak at around 101.2–101.6 eV was not successful. Hence, additional deconvolution of the C1s spectra was therefore necessary.

**Figure 3:**
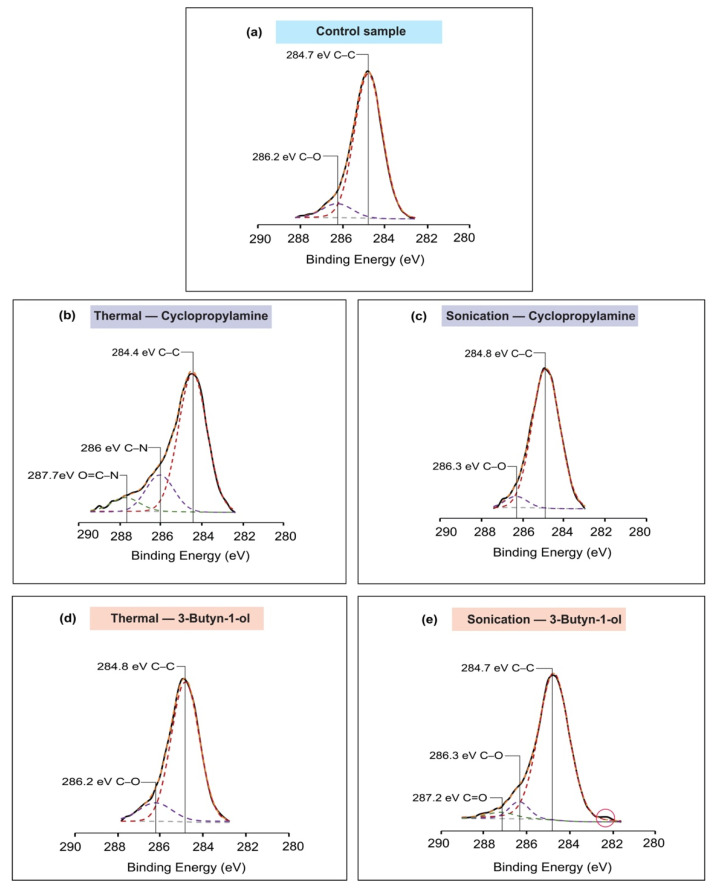
The analysis of the C1s spectra of each sample. (**a**) Control sample; (**b**) cyclopropylamine thermal reaction with C–N and O=C–N linkage type; (**c**) cyclopropylamine sonochemical reaction; (**d**) 3-Butyn-1-ol thermal excitation; (**e**) 3-Butyn-1-ol sonochemical reaction with carbon–carbon and carbon–oxygen peaks. The outlined part suggested that the reaction may have occurred.

The spectrum of the high-resolution C1s spectra of the samples had shown the C–C peaks at 284.4–284.8 eV [56,57,58] and C–O peaks at 286.2–286.3 eV [59,60]. For cyclopropylamine-based thermal reaction, a peak was observed at 286 and 287.7 eV which were, respectively, indicative of a C–N bond [61,62] and O=C–N bond [63,64,65] as presented in Figure 3b. However, for the sonochemical reaction of cyclopropylamine, the absence of C–N bond was another reinforcing proof that the reaction did not take place through the nucleophile NH_2_. On the other hand, by comparing the C1s analysis of 3-Butyn-1-ol thermal to the sonochemical reaction, a peak at the 287.2 eV position (Figure 3e) was successfully fitted for the sonochemical reaction sample and was characteristic of a C=O bond [66,67]. More importantly, as highlighted in Figure 3e, a notch was noticed at about 282.2 eV and this could be representative of Si–C bonding, as described by Wang et al. [68].

**Figure 4:**
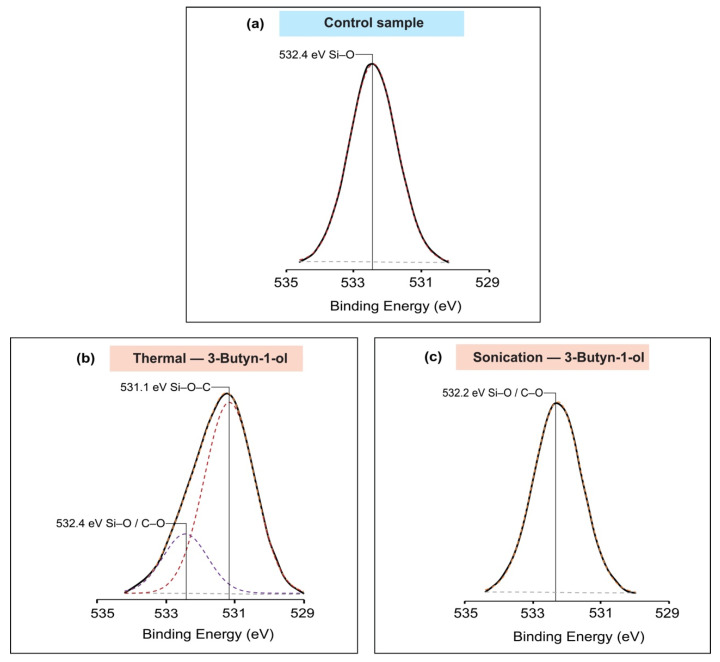
Deconvolution of the high-resolution O1s spectra (**a**) control sample; (**b**) thermal reaction of 3-Butyn-1-ol with two peaks observed at 531.1 and 532.4 eV; (**c**) sonochemical reaction of 3-Butyn-1-ol.

The deconvolution of the O1s spectrum was necessary to identify the nature of the oxygen bonds. A single peak was fitted at a position of 532.4 eV for the control sample, which was representative of Si–O bonding [69]. Furthermore, two peaks were successfully fitted in the thermal reaction spectrum analysis of 3-Butyn-1-ol. As shown in Figure 4b, the smaller peak at the same position of 532.4 eV indicated a Si–O/C–O linkage type while the larger peak fitted at 531.1 eV was representative of Si–O–C bond [45,70]. Comparatively, the deconvolution of the sonochemical reaction sample resulted in a unique peak at 532.2 eV which was suggestive of a Si–O/C–O bonding type [71,72]. These findings further support that the sonochemical-based reaction did not occur through the OH group with the formation of Si–O–C bonds, but rather might have taken place via the alkyne group with the formation of a Si–C bond. The different peaks from the O1s spectra analysis were as displayed in Figure 4.

**Figure 5:**
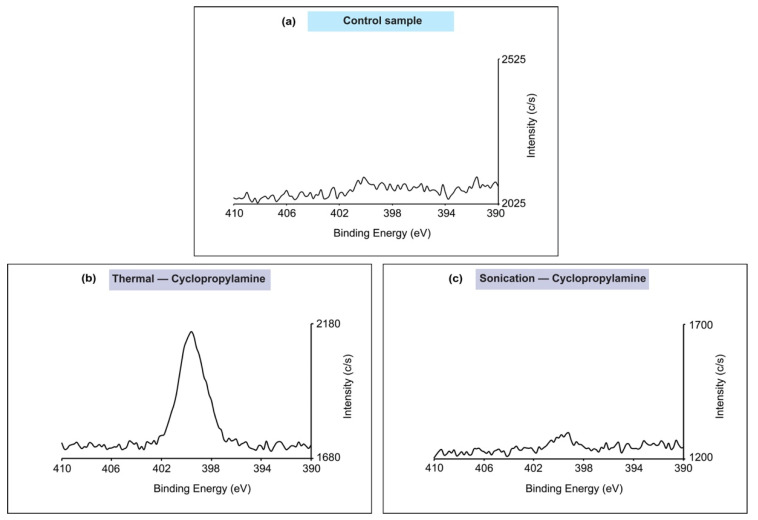
N1s spectra analysis for (**a**) control sample; (**b**) cyclopropylamine thermal reaction; (**c**) cyclopropylamine sonochemical reaction. Both samples (**a**,**c**) showed relatively flat spectrum while the thermal reaction of cyclopropylamine produced a significant peak at around 399 eV.

The high-resolution N1s spectra studies were also important to comprehend the details of nitrogen bond formation. To better correlate the relative nitrogen levels among the samples, the intensity of the spectrum was kept consistent for each of the samples. As shown in Figure 5a, a relatively flat spectrum was observed for the control sample, which was expected since the surface did not undergo any modifications. More interestingly, when comparing the thermal reaction of cyclopropylamine (Figure 5b) to the sonochemical reaction (Figure 5c), a significant peak was noticed at about 399 eV for the thermal reaction while the sonochemical reaction sample showed a plain spectrum without relevant peaks. Based on the literature [73,74], the peak positioned at about 399 eV was found to correspond to the formation of Si–N bonds, thus further confirming that the reaction had occurred through the nucleophile NH_2_. On the other hand, the observation of the flat spectrum for the sonochemical reaction provided further evidence that the sonochemical reaction did not induce any chemical reaction of cyclopropylamine.

The assignments discussed in this study, along with their corresponding binding energy, FWHM and area under the peak, have been summarized in Table 1. The different peaks described in this table were in good agreement with the formation of corresponding chemical bonds as documented in the literature. The combined analysis of this information was substantial in our effort to understand the different types of chemical bonding.

## 4. Discussion

The data acquired through the contact angle measurements in conjunction with the AFM studies and XPS analysis were necessary in our attempt to describe how the directionality of the reaction had occurred. However, prior to discussing the outcome of the reactions in this work, it was important to describe the basic mechanisms that might have taken place during the sonochemical reaction. The underlying mechanism of sonochemical reaction has been discussed in literature in the past by various groups providing contradictory arguments, and this consequently demonstrated the complexity in attempts to elucidate the precise mechanism during sonochemical reactions. Ando et al., were the first to suggest that sonochemical reaction may affect nucleophilic substitutions outcomes [75]. They reported that during sonochemical reaction, waves from ultrasound may accelerate the nucleophilic attack of the alumina surface by the leaving group, hence resulting in a nucleophilic substitution event. This initial study was the first to propose a reaction mechanism that could occur during the sonochemical activation process. Luche et al., subsequently attempted to digress sonochemical reaction in different environments by discussing the concept of ‘true’ and ‘false’ sonochemical reaction [76]. In their context, ‘true’ sonochemical reaction was described as a reaction in which acoustic cavitation creates a coordinated unsaturated element and free radicals to influence the chemical outcome of the reaction in homogeneous solution, while the outcome of the ‘false’ sonochemical reaction was due to the mechanistic impact of sonochemical reaction in a heterogeneous solution. The notion that a sonochemical-based reaction is merely a mechanistic rather than a chemical operation was also discussed by Kegelaers et al. [77] who, after repeating the same series of experiments as described by Ando et al. [75], came to the different conclusion that sonochemical reaction served as a mechanistic approach akin to those from extensive stirring that shortens the reaction time [78] but by itself did not induce a chemical change. Despite various examples of ‘true’ [79,80,81] and ‘false’ [82,83] sonochemical reactions in literature, which so far remain as the most common explanations, it is necessary to acknowledge that the exact mechanism of sonication chemistry has not yet been clearly elucidated [84].

Prior to this work, we had demonstrated that during a thermal excitation reaction, cyclopropylamine reaction to silicon hydride surface occurred via the NH_2_ nucleophilic group to form Si–N bonds [47] while 3-Butyn-1-ol reacts to silicon hydride through the OH group to form a Si–O–C bond [45]. These preferences were largely due to the fact that the electronegative domain from the nucleophile tends to offer precedence over unsaturated carbon groups. The main question to address in this study was to determine the reaction directionality of these bifunctional molecules during the sonochemical process. In the cyclopropylamine-based sonochemical reaction, the absence of Si–N bonds observed in the Si2p spectrum (Figure 2c), along with the lack of surface reorganization due to oxidation as shown in the AFM studies (Figure 1b), could indicate that the nucleophilic group did not react aggressively to the silicon hydride surface. This non-reaction of the nucleophile on the silicon hydride surface can be explained by the effect of acoustic cavitation, whose violent collapse could continue to break the coordinative bonds upon surface formation. In fact, the mechanical effect of sonochemical reaction has shown to induce coordination bond breaking, as reported by Paulusse et al. [85]. The continuous cycle of bond formation and breakage could explain the non-reaction observed during sonochemical reaction. Moreover, during sonochemical reaction, despite the fact that the explosion of bubbles due to acoustic cavitation produces a high temperature, such a high temperature lasts only a few microseconds, as described by Suslick [29], and may not be sustainable enough to initiate a chemical reaction of the nucleophilic group. The absence of sustained heat generated during the sonochemical reaction might also lead the silicon hydride surface to repel the cyclopropane nucleophilic group from the surface.

**Figure 6:**
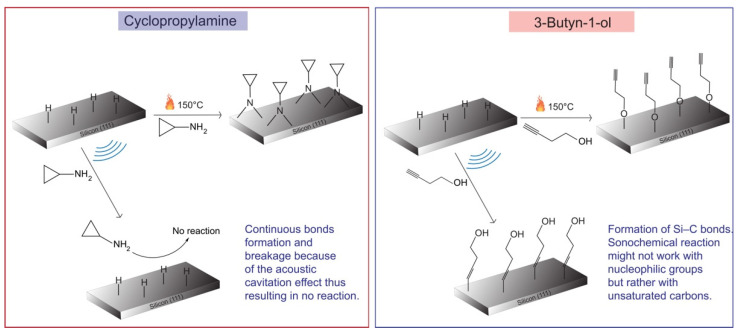
Proposed schematic of the reaction pathways for the grafting of cyclopropylamine and 3-Butyn-1-ol during high-temperature thermal and sonochemical reaction.

On the other hand, for the 3-Butyn-1-ol-based sonochemical reaction, the silicon hydride surface reacted to the unsaturated carbon of the molecule to form Si–C bonds and this was as observed in Figure 3e. The mechanism of this reaction with the silicon surface still remains complex; however, some elements of responses can be found in the work reported by Korgel’s group [86]. They described that during hydrosilylation reaction at room temperature, there is a mechanism where the nucleophilic group correlates to the silicon surface to increase the reactivity of the hydrogenated silicon surface, thus leading to the extension of the Si–H bond. The extended Si–H bond can then react with other unsaturated carbons of the same molecule. From these analyses, the final reaction pathway of both cyclopropylamine and 3-Butyn-1-ol can be described as illustrated in Figure 6.

## 5. Conclusions

Sonochemical reaction is an interesting activation method for surface characterization because it offers a good reaction rate [87] as well as the possibility to understand the influence of ultrasound on chemical bond formation on planar silicon surfaces. In this report, we carefully examined the reaction preferences of nucleophiles and unsaturated carbon in bifunctional molecules, and we subsequently proposed a reaction pathway for the grafting of the molecules based on the analysis of the experimental results of both reactions. The results from thermal reaction serving as our guide had suggested direct nucleophilic addition to the silicon hydride surface, thus resulting in the formation of silicon nitride bonds on the surface, while the sonochemical activation with the effect of acoustic cavitation does not induce a similar reaction profile due to the hypothesized process of constant dative bond formation and breakage as a result of acoustic cavitation on the surface. In contrast, with the 3-Butyn-1-ol which has an alkyne and a nucleophile end, the sonochemical reaction preferentially takes place through the alkyne group of the molecule that in turn stabilizes the surface grafting even during the process of acoustic cavitation. Furthermore, observations from our thermal reaction also support the claim that the reaction inversely occurs through the hydroxide group. These findings are important as they provide more insights into the underlying mechanism of sonochemical reaction and could further open up an avenue of research to understand the behavior of nucleophile and alkyne during this process.

## Figures and Tables

**Table 1:** Representative summary of peak assignments reported in this work.

Reaction Model			Binding Energy (eV)	Assignment	FWHM	Area under the Peak
Control Sample		Si2p	99.1	Si2p _3/2_	0.9	6997.69
			99.8	Si2p _1/2_	0.93	4361.18
			102.7	Si–O_x_	2.02	3225.73
		C1s	284.7	C–C	1.53	4289.18
			286.2	C–O	1.65	458.30
		O1s	532.4	Si–O	1.67	17,751.58
Cyclopropylamine	Thermal reaction	Si2p	98.7	Si2p _3/2_	0.91	2767.26
			99.3	Si2p _1/2_	0.98	1855.55
			101.9	Si–N	2.17	3135.52
			103	Si–O_x_	1.55	1730.05
		C1s	284.4	C–C	1.64	3814.62
			286	C–N	1.62	1000.39
			287.7	O=C–N	1.66	439.55
	Sonochemical reaction	Si2p	99.1	Si2p _3/2_	0.88	6271.15
			99.8	Si2p _1/2_	0.95	4823.99
			102.6	Si–O_x_	1.89	1720.59
		C1s	284.8	C–C	1.57	2897.68
			286.3	C–O	1.27	185.57
3-Butyn-1-ol	Thermal reaction	Si2p	101.4	Si–O–X	1.64	3279.78
		C1s	284.8	C–C	1.54	3182.46
			286.2	C–O	1.78	469.47
		O1s	531.1	Si–O–C	1.72	10,290.74
			532.4	Si–O/C–O	1.53	2879.55
	Sonochemical reaction	Si2p	99	Si2p _3/2_	0.95	5912.40
			99.7	Si2p _1/2_	1.05	3856.94
			102.6	Si–O_x_	1.96	1979.99
		C1s	284.7	C–C	1.68	4057.23
			286.3	C–O	1.07	280.74
			287.2	C=O	1.46	140.44
		O1s	532.2	Si–O/C–O	1.68	10,898.10

## Data Availability

Not applicable.

## Sample Availability

Samples of the compounds cyclopropylamine and 3-Butyn-1-ol are available from the authors.
